# People Counting Using YOLO-Based Detection and Clustering for a Mobile Robot

**DOI:** 10.3390/s26154664

**Published:** 2026-07-23

**Authors:** Kamil Gomulka, Piotr Wozniak, Tomasz Krzeszowski

**Affiliations:** 1 Faculty of Electrical and Computer Engineering, Rzeszow University of Technology, al. Powstancow Warszawy 12, 35-029 Rzeszow, Poland; k.gomulka@prz.edu.pl (K.G.); p.wozniak@prz.edu.pl (P.W.); 2 Doctoral School, Rzeszow University of Technology, al. Powstancow Warszawy 12, 35-029 Rzeszow, Poland

**Keywords:** people counting, people detection, person re-identification, YOLO, visual clustering, mobile robots

## Abstract

People counting is one of the key tasks in intelligent monitoring systems. However, accurately counting people in dynamic environments can be extremely challenging. This is especially true in mobile robot applications, where challenges such as a moving camera, varying conditions, and a limited field of view due to environmental obstacles arise. In such scenarios, the people counting task primarily involves visually detecting and grouping individuals to determine the total number of unique people. This paper presents a people counting algorithm based on visual people detection and clustering. The method utilizes the You Only Look Once (YOLO) detector to identify the bounding boxes of detected individuals and extract features from their corresponding regions of interest (ROIs). Additionally, the dimension of the extracted features is reduced using an encoder and clustered to distinguish individuals, with the number of clusters serving as an estimate of the number of people. The method was tested on a dataset containing 45 independent sequences with a total of 19,350 RGB images, complete with metadata for people detection and re-identification. This dataset encompasses various settings, particularly scenarios featuring mobile robots moving and capturing frames in indoor environments. The experiments demonstrate the effectiveness of the proposed method in different configurations. The best results were achieved using the YOLOv10n detector combined with K-means or SK-means clustering, yielding a Mean Absolute Error (MAE) of 1.11. The proposed encoder-based method significantly reduces clustering time and operates effectively within the limited resources available on mobile robotic platforms such as the Jetson Nano.

## 1. Introduction

Human detection and counting constitute one of the fundamental tasks of surveillance robots. Advances in research on robotic platforms, computer vision, and sensor technology have greatly enhanced counting accuracy and real-time performance. In the current literature, many methods for people counting have been proposed, ranging from non-vision-based solutions to computer vision techniques using deep learning. These methods leverage a variety of sensors, including cameras, depth sensors, and radar-based technologies. For example, the use of RGB-D cameras, such as Kinect, serves as the basis for many human counting systems [1]. Another approach is to use devices, where an impulse radio ultra-wideband radar is employed to analyze signal patterns and count people in real-time, enabling accurate counting even in challenging conditions [2]. A significant area of research focuses on modern techniques that rely on vision methods such as YOLO [3,4]. These methods achieve high accuracy in diverse lighting conditions, various place layouts, and occlusions [5]. Furthermore, the integration of tracking and re-identification algorithms has further improved these systems, enabling them to track people across different camera views [6,7,8]. Recent advancements have introduced Vision Transformer (ViT) as an alternative to Convolution Neural Network (CNN) methods, using the self-attention mechanism to improve feature extraction and robustness [9]. These approaches provide valuable information and further enhance the effectiveness of current systems, such as personalized services and security [10,11]. The application of object counting and tracking is not limited to public spaces and urban surveillance. Robots, especially those involved in agriculture or retail, have also integrated counting systems for monitoring and stock management [12,13]. The completion of the tasks mentioned above using mobile robotic platforms presents a particular challenge in ensuring reliable operation. Mobile robots that move through the environment offer clear advantages over systems with static cameras and sensors, which can only react to the environment in a limited way.

Previous research on people and object counting methods has focused primarily on two main approaches: people counting using static cameras, such as industrial cameras [14,15], and mobile robots equipped with sensors [16]. These systems benefit from stable viewpoints and consistent lighting, which simplifies the detection and counting process. However, counting people using mobile robots introduces unique challenges that consist of constant changes in perspective, dynamic backgrounds, or motion blur. In addition, mobile platforms often operate under varying lighting conditions, limited field of view, and constant occlusions, making tasks such as people detection, identification, and grouping challenging. These factors pose significant challenges. To address them, unlike traditional tracking methods that rely on continuous trajectories and often fail under dynamic robotic viewpoints, we propose a sequence-level clustering framework. By aggregating visual features across the entire image sequence, our approach eliminates the need for strict temporal continuity. This allows the system to robustly count unique individuals even if they are heavily occluded or temporarily leave the field of view, providing a reliable solution tailored for mobile robotic platforms.

To address the research gaps identified in the literature on mobile robot-based people counting, this paper makes the following key contributions:

- Proposing a novel algorithm for visual counting people based on clustering for a mobile robot;
- Incorporating optional modules, including an additional backbone for enhanced features and an encoder to reduce dimensionality;
- Extending the MDDRobots dataset [17] with manually labeled metadata for visual detection, re-identification, and people counting;
- Verifying the proposed method on the dataset containing indoor recordings from real mobile robots;
- Evaluating and comparing the method’s performance across platforms with varying computational capabilities.

The outlined research contributions are systematically realized in the subsequent sections of this paper. Section 2 provides a comprehensive review of the related literature, highlighting the limitations of current vision-based counting methods and establishing the context for our approach. Section 3 details the proposed visual people counting framework, presenting the clustering-based algorithm and the integration of the optional encoder module for dimensionality reduction, which constitute the core methodological contributions of this work. Section 4 introduces the extension of the MDDRobots dataset with manually labeled metadata, directly addressing our data-related contribution. Section 5 presents the experimental setup and results, fulfilling the remaining contributions by evaluating the method’s detection and counting performance across hardware platforms with varying computational capabilities. Section 6 provides a critical analysis of the method’s practical limitations, failure cases under dynamic viewpoints, and the inherent trade-offs between computational efficiency and counting accuracy. Finally, Section 7 summarizes the key findings and highlights the potential of this work for future mobile robotic applications.

## 2. Related Work

### 2.1. Non-Image-Based Approaches

People counting methods can be differentiated into two main categories: image-based and non-image-based approaches. In non-image-based solutions, radio frequency (RF) technologies are frequently used. By analyzing disturbances in the signals, these RF systems can identify the presence of humans. Yuan et al. [18] have shown that with an iterative calibration mechanism, received signal strength indicator-based solutions can achieve high precision even with measurement errors. A different approach utilizing LiDAR was also suggested as part of the sensor-based solutions. Hashimoto et al. proposed a method that uses laser scanners at multiple heights to detect people by tracking their heads and knees [19]. While providing high accuracy, this approach comes with significant drawbacks in the form of sensor cost, which can limit scalability. PIR sensors, on the other hand, offer a more cost-effective solution. Raykov et al. [20] utilized a single PIR sensor and an infinite hidden Markov model to predict room occupancy. Many similar approaches have found success in areas such as public transport applications [21]. However, due to reliance on temperature changes and short transmission range, these systems struggle in outdoor environments.

### 2.2. YOLO-Based Detection

While sensor-based methods can be useful for basic counting tasks, they are less effective in addressing complex, dynamic indoor or outdoor scenarios. One of the key aspects of image-based people counting is the detection of representative visual patterns of humans. To achieve that, a precise location of each present person is necessary. In recent years, solutions that incorporate deep learning techniques have gained significant attention due to their ability to process images. These models are typically trained and evaluated on large datasets that contain annotated images. Examples of popular datasets include COCO [22] and Object365 [23], which provide diverse real-world images with varying lighting and object scales to ensure the robustness of model training. Based on these data, various detection models have been developed, including DETR (DEtection TRansformer), RCNN (Region-based Convolutional Neural Network), and FCOS (Fully Convolutional One-Stage Object Detection) [24,25,26,27], each presenting different advantages for object detection. However, one of the most well-known models in the literature and used is YOLO [28], renowned for its speed and accuracy, making it particularly suitable for real-time applications. YOLO’s versatility has been demonstrated across multiple fields. Vu et al. [29] proposed a real-time system for detecting packaging defects, which uses YOLO to identify defective products on production lines with high precision and speed. It demonstrates method effectiveness in industrial quality control applications. In the field of robotics, YOLO-based tracking has been widely explored for tasks such as counting agricultural products, such as tomatoes [30]. Similarly, Yang et al. [31] demonstrated the use of AD-YOLO combined with MR-SORT to count apples in agricultural settings.

### 2.3. YOLO-Based People Counting

A particular example of using YOLO is visual people detection and counting. The use of this type of universal detector in color images is the basis of many detection and tracking methods [32]. Numerous studies have focused on detecting and counting moving people in different variants of the scene. For example, Ren et al. [5] used a YOLO-based approach to count people in real-time with a static camera, while also considering environmental factors. McCarthy et al. [33] explored video-based automatic people counting for rail replacement bus services, where managing large crowds is crucial. In their research, certain challenges have emerged regarding factors such as lighting and weather, which can affect the accuracy of systems. The cited research focuses on analyzing scenes in which many people are moving around the scene, and counting is performed without the need for individual identification. However, in some cases, it is also important to determine precisely whether a given person will re-enter the scene. The process of person re-identification in counting is crucial in this case, as direct information is lost when an individual leaves the camera’s field of view. Ye et al. [34] proposed an extended variant of people tracking and re-identification implemented using multiple indoor cameras. The referenced works highlight the challenge of detecting, re-identifying, and counting individuals using a mobile robot. In subsequent sections, we provide an in-depth analysis of the technologies employed to address these challenges.

### 2.4. People Re-Identification and Clustering

Visual person re-identification with the support of grouping is an important approach in the literature. This method enables applications with manually annotated training data [35] and serves as the foundation for unsupervised solutions [36]. Fan et al. presented an unsupervised approach to person re-identification, leveraging clustering techniques to match individuals across views [10]. Unsupervised learning uses various machine learning techniques, including deep neural networks such as CNN. Using deep learning and clustering methods, Zhao et al. [37] presented pedestrian retrieval from different cameras without manual annotation. Zhai et al. [38] proposed an extended discriminative clustering technique to improve person re-identification using unlabeled target domain samples. It forms the basis of the current work, emphasizing the critical role of domain adaptation in both re-identification and clustering tasks. Several of the studies mentioned above confirm the importance and feasibility of solving the person-counting problem. Recent advances include also lifelong learning approaches that enable continuous adaptation to new data while preserving previously learned representations [39]. The challenge of counting using mobile robots involves addressing people’s universal re-identification.

### 2.5. Robotics and Real-World Applications

Many of the previously discussed articles lay the groundwork for applications in robotics, as tasks like visual grouping and object counting are essential for robots to understand and interact with their environment. Robots monitor their environment and use images to detect and identify objects. For example, Kejriwal et al. [13] presented a system for counting products specifically designed to evaluate robot-based retail stock. Similarly, Zhang et al. [12] employed deep learning-based algorithms for accurate crop counting in agricultural robots. One of the primary challenges in using such systems involves the diversity of robot platforms and their types of movement, such as driving or flying drones [40]. Counting people with mobile robots presents several challenges, mainly due to the change in camera position, which adds further complexity. This requires continuous visual detection and re-identification [41,42], further complicating the task. Unlike counting facilitated by static cameras supplemented with numerous sensors [43,44], the capabilities of mobile robots are often limited, requiring an increased workload to adapt solutions to these constraints. In summary, while existing vision-based people counting methods show promise, a research gap remains regarding mobile robotic platforms. Current approaches often assume static camera viewpoints, which do not account for the challenges of constant perspective changes, motion blur, and frequent occlusions inherent to dynamic environments. Moreover, there is a need for counting frameworks that balance accuracy with the computational constraints of embedded edge devices. This paper addresses these gaps by proposing a counting framework designed to be adaptable to various robotic platforms, with a focus on optimizing computational efficiency through dimensionality reduction.

## 3. Proposed Method

**Figure 1:**
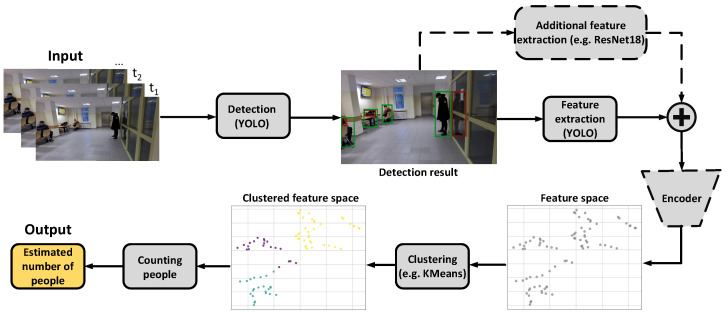
General schematic of the proposed visual people counting method. The architecture integrates a YOLO-based detection and feature extraction stage, followed by an encoder block for dimensionality reduction. The final count is derived through sequence-level feature clustering, ensuring consistency across multiple frames.

The proposed method is structured as an integrated pipeline that transforms per-frame object detections into a sequence-level count. Although individual components, such as YOLO and clustering, are established techniques, our methodological contribution lies in their systematic integration: we utilize optional encoder-based dimensionality reduction to compress diverse feature representations into a compact space, enabling consistent sequence-level grouping. A general schema of its processing stages is shown in Figure 1, while Algorithm 1 presents the pseudocode of the proposed method. In the first step, the method processes a sequence of RGB images recorded from a moving camera. Each image is fed into the YOLO detection model, adapted for, among others, human detection in order to identify bounding boxes of this class. Additionally, within the detection stage, filtering is performed based on the predicted probability, and the size of the bounding box, which delineates the region of interest (ROI). As a result, detection bounding boxes are retrieved from the first stage of the YOLO network. Features are then extracted from its backbone, which is pre-trained on the COCO dataset. For the purposes of this method, specific layers of the network were selected to provide a strong representation suitable for the problem. Knowing the ROI from each frame, a representative feature is obtained for the detected area. The features are tensors, with their size depending on the detected bounding box and the dimension of the layer. To reduce these multidimensional features into a single feature vector, adaptive average pooling is applied, aggregating spatial information from the entire feature map into a single value per channel and producing a compact representation of the ROI. An optional step is to extend the model with additional features from another network, similar to the one extracted from YOLO. As a result, extended features are created by concatenating both representations. Afterward, a trained encoder block is used to reduce the dimensionality of the feature vector and improve its representation. The features, in the form of vectors, are then gathered from the detections for every image. Since the method is based on sequences of an arbitrary number of frames, it is not necessary to predict the number of people in every frame. The collected features are used to group the data and determine the optimal number of clusters. The method considers both the maximum number of people observed in a single frame and the predicted cluster assignments. Notably, the final estimate is derived from the entire image sequence, with all frames being analyzed jointly to maintain consistency between frame-level observations and the resulting clustering. Consequently, the proposed approach should be regarded as a sequence-level people-counting method rather than a strict frame-by-frame counting system. The final estimate is obtained after aggregating features from multiple frames and jointly processing them during clustering. It is worth emphasizing that this counting result can be obtained for sequences of any length, making the method flexible for various scenarios. Note that the entire counting process relies exclusively on the predictions generated by the YOLO detector. Ground-truth information is never utilized during the counting process; it is only used for performance evaluation and calculating metrics presented in the Section 5.
**Algorithm 1** Visual People Counting Using YOLO and Feature Clustering**Require:** Image sequence $S=\{I_{1},\ldots ,I_{n}\}$, YOLO detector *D*, optional backbone *B*, optional encoder *E***Ensure:** Estimated number of people *N*  1:Initialize feature collection F←[]  2:Initialize the maximum number of detected people in image frame M←0  3:**for** each image I∈S **do**  4:      Detect person bounding boxes $P=\{p_{1},\ldots ,p_{k}\}$ using *D*  5:      Filter detections by confidence and minimum ROI size  6:      M←max(M,|P|)  7:      **for** each detection p∈P **do**  8:            Extract the ROI feature map $F_{m}$ corresponding to *p* from selected YOLO layers  9:            Obtain feature vector $f\leftarrow \mathrm{adaptive\_average\_pooling}(F_{m})$10:            **if** additional backbone *B* is used **then**11:                   Extract backbone feature vector $f_{B}$ for ROI *p*12:                   $f\leftarrow Concat(f,f_{B})$13:            **end if**14:            **if** encoder *E* is used **then**15:                   f←E(f)16:            **end if**17:            Append *f* to *F*18:      **end for**19:**end for**20:**if** the selected clustering method requires the number of clusters **then**21:      Determine the optimal number of clusters by clustering the feature set *F*22:**else**23:      Cluster feature set *F* using the selected clustering method24:      Let *K* be the number of obtained clusters25:**end if**26:N←max(K,M)27:**return** *N*

### 3.1. YOLO-Based People Detection

The proposed method is based on the YOLO architecture, which is specifically designed for real-time object detection. YOLO is a state-of-the-art convolutional neural network that is known for its ability to predict multiple bounding boxes and their corresponding class labels from an image in a single forward pass. Unlike traditional methods [26,45,46], which freely process areas of interest, YOLO splits the image into a grid and assigns a bounding box and class probabilities to each grid cell. This makes it highly efficient and suitable for fast detection tasks. The YOLO architecture consists of three main modules. First, the backbone, typically a convolutional neural network, is responsible for feature extraction. In this step, the image is transformed in each subsequent layer into a high-level representation. Different versions of YOLO may utilize different backbones, such as DarkNet53 [47], CSPDarknet53 [48], or EfficientRep [49]. The second main component is the neck, responsible for processing the extracted features, enhancing representation, and passing them to the last part, the detection head. Finally, the detection head processes the extracted features and generates final bounding boxes and class predictions. It uses anchor boxes and parameters, such as objectness scores, to refine detection and improve final accuracy.

**Figure 2:**
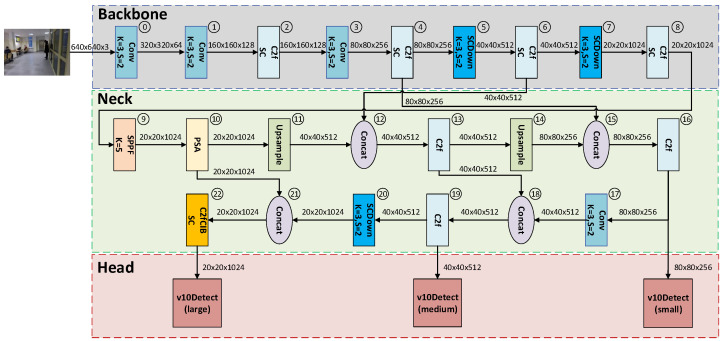
Architecture of the YOLO version 10 nano (YOLOv10n) model.

The constant development of the YOLO results in gradual improvements in each new proposed version. A recent model integrated in our method is YOLOv10, which further optimizes both speed and accuracy [50]. The key advancement was the removal of the non-maximum suppression during training. Instead, a novel dual-assignment strategy is used, which allows the model to handle overlapping predictions more efficiently, thus improving inference speed. YOLOv10 introduced a lightweight classification head, reducing computational complexity. In addition, large-kernel convolutions and partial self-attention modules are used to further enhance the feature extraction step and allow the model to better distinguish objects in complicated scenes. The presented regimen results in noticeable improvements in the model, making YOLOv10 suitable for precise and fast object detection tasks. In this work, we used several YOLOv10 models of varying sizes, including YOLOv10n. It is the most lightweight variation with the architecture presented in Figure 2. In the first part, a series of convolutional layers and C2f modules with a shortcut connection (SC) progressively extract features while reducing spatial dimensions. Two SCDown operations perform downsampling, increasing the number of channels to enhance feature richness. The neck further refines these features through SPPF and PSA modules, while multiple upsampling and concatenation operations ensure effective multiscale fusion. Furthermore, the C2fCIB with SC layers contributes to strengthening feature propagation. The detection head consists of three v10Detect modules that operate on different scales, allowing efficient localization of small, medium, and large objects.

In terms of the people counting task, the YOLOv10 models are used both to extract features and to detect. During the estimate, the model generates several bounding boxes with affiliated confidence scores and class labels. Since the original model was trained to detect 80 object categories, but our focus is solely on the person class, predictions are filtered accordingly. In addition, to refine these predictions, a thresholding strategy is employed: a bounding box with a confidence score is maintained only if it exceeds a certain value. This ensures that only high-confidence detections are considered, reducing the risk of false positives. Additionally, to further filter irrelevant detections, the bounding boxes smaller than a specific size are abandoned. It ensures that only meaningful human detections are kept.

### 3.2. Feature Extraction

To collect the representations of people in the detector model, a feature extraction mechanism has been incorporated into the proposed method. For this purpose, callbacks were used to access the outputs of selected internal layers of the YOLOv10 model, enabling the capture of complex features without modifying the model architecture. For the YOLOv10n architecture shown in Figure 2, features were extracted and tested in separate variants. Two layers, selected to evaluate the effectiveness of multi-path feature fusion, correspond to the outputs of block 15 (80×80×256) at the input of the C2f convolutional block, and block 21 (20×20×1024) at the input of the C2fCIB_SC block. The features extracted from these layers are first pooled using adaptive average pooling and then concatenated to form the final ROI representation, resulting in a 1280-dimensional vector. Additionally, an unfused representation was extracted from a layer positioned before the block 20 concatenation, enabling direct comparison with the fused counterparts. After detecting bounding boxes, the corresponding areas on the feature map are extracted based on their spatial locations. This step is followed by adaptive average pooling, which condenses the features within each bounding box into a fixed-size vector representation.

Beyond the YOLO model, the method also supports feature extraction from external backbone networks such as ResNet-18 or MobileNetV3. In this scenario, each detected region is fed into the backbone and features are harvested from its last layer. Similarly to how we extract features from YOLO, adaptive average pooling is used to compress spatial feature maps into a fixed-size feature vector. Both the YOLO-based and the backbone features are concatenated to form a joint representation for the final prediction. This approach provides flexibility, allowing the use of different feature extractors with diverse capabilities, although it comes with an increase in computational complexity.

**Figure 3:**
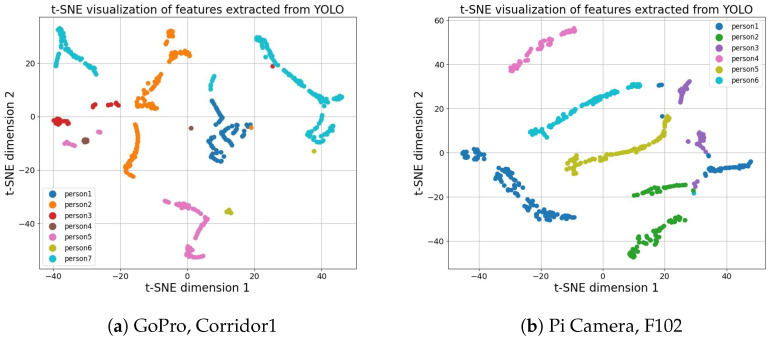
Example t-SNE visualization of features extracted from dataset subsets.

Examples of extracted features are visualized in a 2D space using t-SNE, as shown in Figure 3. From the visualization presented, observations can be made about distinct groupings of features that are separated, indicating a different person. However, challenges arise in cases where the features of a specific person (e.g., person 7 in Figure 3a and person 1 in Figure 3b) are further apart because the person reappears with a different background and perspective. This highlights the difficulty of consistently capturing the same person and properly re-identifying them in each successive frame.

### 3.3. Encoder

**Figure 4:**
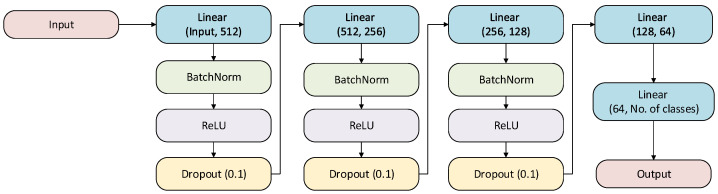
Encoder 64 architecture during training phase.

To further process the extracted features and reduce their dimensionality while retaining important information, we introduce a compact encoder module. The objective of this step is to transform the high-dimensional vectors produced by YOLOv10, with optional extension to features from other backbones, into 32- or 64-dimensional descriptors. The encoder consists of a sequence of fully connected layers, where each is followed by batch normalization, ReLU activation, and dropout, which improves generalization and helps mitigate overfitting. The general structure of the encoder, which reduces the dimensionality to 64, is shown in Figure 4. For the 32-dimensional variant, the architecture is extended at the end with an additional fully connected layer with its associated batch normalization, activation, and dropout stages. Initially, a classification head is used during training to ensure that the latent representations capture distinct features for approximately 20 different individuals, and each class represents a different person. This ensures that useful and discriminative features are preserved. However, once training is complete, the encoder operates solely for dimensionality reduction, without the need for classification. Training is performed on the GoPro subset of the dataset to provide a diverse set of instances that will be representative of the people counting task. The Adam optimizer was used with an initial learning rate of 5e-5, a batch size of 32, and a total of 200 epochs. The model is initially trained to minimize the cross-entropy loss, ensuring that the latent space representations are informative and suitable for subsequent tasks, such as visual clustering. Cross-entropy loss internally applies softmax activation over the output classes, so no additional activation function is required at the model output. After training, the encoder is capable of generating low-dimensional feature vectors from input features extracted by YOLOv10 during detection.

### 3.4. Clustering

**Figure 5:**
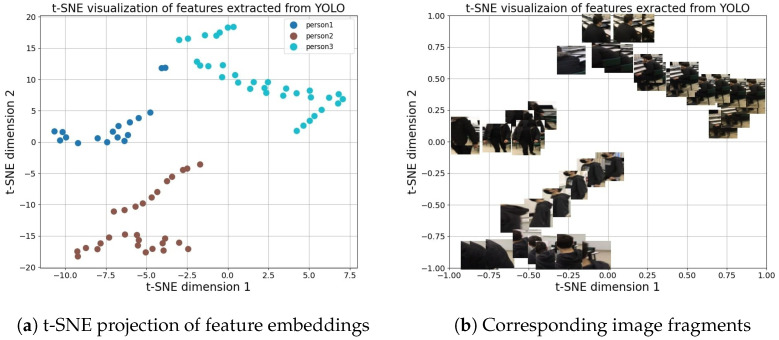
Example of visualization of extracted features from the iPhone subset.

Following the detection of people and the extraction of features, the next step involves clustering. The main goal is to group the data based on their similarity to the detected people, enabling estimation of the number of unique humans present in a given sequence. Two distinct charts are presented, one presenting the position in feature space of each extracted feature from detection, the other more clearly showing which part of the image each feature point represents. Based on observations, in some sequences, the features obtained from the detection exhibit greater separability for the person identification problem. As shown in Figure 5a, in a sequence containing three labeled individuals, features and their distribution correspond to this number. However, some features may still appear further apart from the main clusters. A closer look at Figure 5b, where each point corresponds to a representative part of the image, illustrates how similarities occur depending on the unique characteristics. At the same time, the background can falsely influence the position of certain features.

Several clustering methods can be applied to group features for counting purposes. Some were selected based on a review of the literature and best practices. The K-means [51] and the spherical K-means (SK-means) [52] were explored to partition the feature space. Since the number of clusters was previously unknown, an estimation technique was applied to identify areas where adding more clusters no longer significantly improved separation quality. Spherical K-means, leveraging cosine similarity instead of Euclidean distance, can prove particularly useful when working with high-dimensional feature vectors. Another tested clustering method was the mean-shift [53]. It was evaluated for its ability to detect clusters without requiring a predefined number. By iteratively identifying dense regions in feature space, it offers flexibility but requires careful tuning of the bandwidth parameter to balance cluster cohesion. The last implemented clustering algorithm was DBSCAN [54]. It offers robustness in handling arbitrary cluster shapes and filtering out noise. The threshold for how close points must be to each other to be considered related, as well as the minimum number of such nearby points required to define a group, were adjusted to handle varying feature distributions and effectively distinguish between valid clusters and outliers.

## 4. Dataset

**Figure 6:**
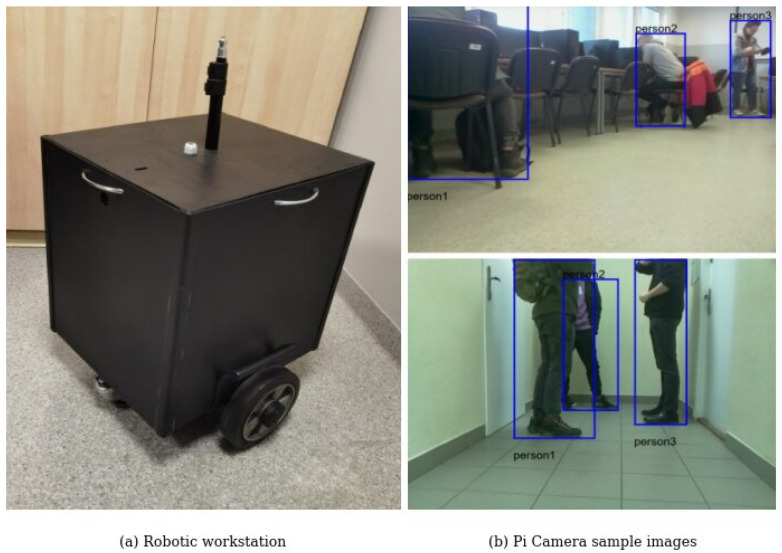
Overview of the experimental configuration and dataset samples including (**a**) a mobile workstation equipped with a mounted Pi Camera, and (**b**) sample images captured during the experiment showing ground truth labels in blue.

The dataset used in this study is part of the MDDRobots collection [17], which also includes a sequence of environments without people. In this paper, we specifically focus on sequences that contain a human presence comprising five distinct subsets. Each subset consists of nine sequences from a variety of indoor places. These sequences were captured with different cameras, distinguished by parameters and recording methods. Two of the subsets were captured by moving mobile robots. The wheeled robot, shown in Figure 6a, had a Pi Camera mounted at a height of approximately 60 cm above the surface. The other robot, Robot Nao, had an ASUS Xtion PRO Live camera mounted on its head, also positioned at a height of around 60 cm. The remaining three subsets were recorded using manually operated cameras. The devices used included GoPro Hero 5 Black, iPhone 6s, and Huawei P40 Pro. The cameras were capable of recording at 30 or 60 FPS, depending on the device. However, because of the small changes between consecutive frames, the sequences were downsampled to reduce redundancy and data volume. The mean FPS (mFPS) values for each subset, after downsampling, are presented in Table 1. In total, the dataset comprises 19,350 RGB images in PNG format. The complete source code (v1.0), including annotations and the full experimental pipeline, is publicly available on GitHub at https://github.com/GomulkaK/PeopleCounting (see [55] for details).

In addition, information on the average number of ground truth bounding boxes (number of persons) per sequence is presented in Table 2. The comparison offers a detailed overview of each subset and its specific sequences. It highlights differences in both the average number of bounding boxes per subset and in individual rooms. On the one hand, the Xtion subset is characterized by the least average number of detected persons per image, which may also result from a lower number of registered individuals. On the other hand, the P40Pro subset has more than two people in the frame on average. It is important to mention that the recordings consist of some frames in which no people are present, which reduces the average.

The images used have been enriched with additional metadata. Human detection labels were manually created for each of the 45 independent sequences extracted from the dataset. These labels included bounding boxes assigned not only to the general people class but also to individual persons, enabling the dataset to be utilized for detection and re-identification tasks. An example annotation is shown in Figure 6b. The presented dataset demonstrates significant challenges and is a valuable area for further research. Images captured by mobile robots moving around rooms are often difficult to detect, re-identify, and count people. The dynamic pose of the camera, changing lighting conditions, environmental equipment, robot movement, and environmental elements contribute to the increased complexity, making accurate predictions more challenging.

## 5. Experiments

To validate the proposed method, a series of experiments was conducted to assess its effectiveness. The evaluation mainly focused on assessing people detection and counting performance, along with analyzing the execution time of the detection and counting algorithms on the different platforms. In addition, different variants of the method were tested and compared to assess the impact of changes in parameters, differences between subsets, and additional features of the neural network model.

### 5.1. Experiment Setup

The proposed method was tested with various computational devices: a mobile workstation (laptop), a workstation (computing platform), and a Jetson Nano minicomputer (embedded system). For the mobile workstation, the operating system used was Windows 11, and for the other two platforms it was Ubuntu. Detailed information on each unit is presented in Table 3. To evaluate the different variants of the method, the workstation was used due to its superior computational power. The speed and performance of phase-specific time were benchmarked across three configurations, providing a comprehensive evaluation of different computational resources. This approach resulted in an analysis that exhibits a trade-off between speed, accuracy, and hardware capabilities required for real-world applications.

On the considered platforms, PyTorch was used as a deep learning framework, ensuring compatibility with the available CUDA version for GPU acceleration. The installed versions of Python, PyTorch, and CUDA varied according to the hardware configuration. The workstation, running Ubuntu 22.04, utilized Python 3.10 with PyTorch 2.2 and CUDA 12.4. The mobile workstation onboard the Pi Camera robot operated with Python 3.8 and CUDA 11.8, maintaining compatibility with its RTX 3060 GPU. Jetson Nano uses a Python 3.8 environment with PyTorch 1.13 and CUDA 10.8, tailored to the current version of the hardware.

### 5.2. Evaluation Metrics

In this study, we evaluate visual detection and people counting. The first task, detection, involves comparing predicted bounding boxes with ground truth annotations. To assess the similarity between these regions, the Intersection over Union (IoU) is used [56]. It measures the overlap between two regions by dividing the area of their intersection by the area of their union. The second task, people counting, measures the difference between the predicted and actual number of people present in the scene.

For people detection, we use both the F1-score and the average precision (AP). For the F1-score, a detection is considered correct if the IoU exceeds a threshold of 50%. The F1-score is a harmonic mean between precision and recall [57], as shown in (3). Precision is the number of correctly predicted bounding boxes (TP) divided by the total number of predicted bounding boxes, as defined in (1), while recall measures the proportion of correctly predicted boxes out of all ground truth boxes, which is shown in (2). These two metrics are combined in the F1-score, which ensures that the model is not overly biased toward minimizing false positives (FP) or false negatives (FN). The second important metric is based on AP, which represents the area under the precision–recall curve [56]. It provides a summary of the precision across various recall levels, which is calculated as presented in (4). In practice, to evaluate a model across multiple classes, a mean average precision (mAP) is used, which is simply an average of AP across different classes. In our case, we focus only on a single class; therefore, both metrics will be indistinguishable:

(1) $$P=\frac{TP}{TP+FP}$$

(2) $$R=\frac{TP}{TP+FN}$$

(3) $$F1\text{-}score=2\cdot \frac{P\cdot R}{P+R}$$

(4) $$AP=\int_{0}^{1}P\cdot R\;dR$$

Depending on the assumed IoU threshold, there are different variants of average precision. The basic version, AP50, uses an IoU threshold equal to 0.5, and only accounts for detection as true if it exceeds that value. A more restrictive version, which is also used in our study, is AP50:95. This variant calculates the average precision across a range of IoU thresholds, from 0.5 to 0.95, with a step size of 0.05, providing a more comprehensive evaluation of detection performance across varying levels.

Finally, we used a mean absolute error (MAE) to find the discrepancy between the estimated and actual numbers of people. It measures the average of the absolute differences between predicted values (${\hat{y}}_{i}$) and actual values ($y_{i}$) across multiple instances, with *n* representing the total number of sequences evaluated. This metric provides a simple and interpretable indication of how far the predictions are from the ground-truth values. A lower MAE indicates a more accurate prediction, reflecting a minimal deviation from the actual count, and is calculated as shown below:

(5) $$MAE=\frac{1}{n}\sum_{i=1}^{n}\left|y_{i}-{\hat{y}}_{i}\right|$$

### 5.3. People Detection

**Figure 7:**
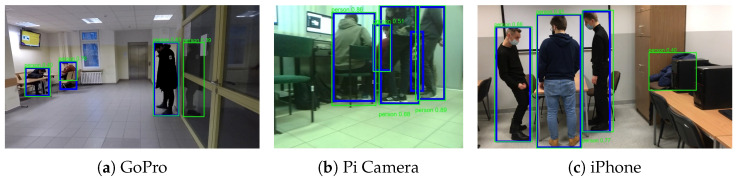
Examples of people detection errors encountered in YOLOv10, where blue boxes represent ground truth labels and green boxes indicate detections, resulting from (**a**) reflective surfaces, (**b**) visual occlusions, and (**c**) the presence of objects such as clothing.

In the first step, which involves detecting people, various popular models were tested. Both CNN-based methods [38,48,58,59,60,61,62], and newer transformer-based approaches [27,63] were compared. Multiple YOLO variants were included in the comparison. These models range from lightweight versions, such as the nano variant with fewer parameters, to larger ones, such as YOLOx. To ensure a fair evaluation, models of medium size were selected from each YOLO family. For each model, the objectness score threshold was individually selected to optimize the F1-score. A higher threshold (e.g., 0.9) was chosen for models like DETR and Faster R-CNN, while lower values were more effective for YOLO-based architectures. Table 4 summarizes the backbone architectures, the number of parameters, the input resolutions, the detection results, and the inference times of the evaluated models. Depending on the variant of the network, the original images were resized to the corresponding input resolution before inference. Upon comparison using both AP and F1-score metrics, the best performance was observed with the RT-DETR-L model, which achieved the highest values in both AP50 and AP50:95, along with an F1-score of 85.87%. However, the processing time for each detection was notably longer, taking twice as long as the YOLO models. To assess the trade-off between accuracy and computational complexity for mobile robotic applications, different variants of the YOLOv10 model were further evaluated. Given that the difference in metrics, particularly the F1-score, was only marginal, around one to two percentage points, and considering the significant advantage in processing time for the YOLO models, the YOLOv10m method was ultimately selected for the task as the baseline. During tests, not only were typical object detection metrics and times calculated, but several specific examples of problematic scenarios were also identified. These situations are illustrated in Figure 7. In particular, as shown in Figure 7a, certain reflective surfaces posed a challenge, often resulting in incorrect additional detections. In such cases, the detector mistakenly identifies a reflection as a person. Additionally, as seen in Figure 7b, in the presence of occlusion, from the Pi Camera perspective, several individuals were not correctly detected. Specific objects that can resemble people or parts of them, like clothes, as demonstrated in Figure 7c, also ended up being difficult for the detector to ignore.

### 5.4. People Counting

The proposed method was thoroughly evaluated to assess visual people counting, taking into account various parameters. The extracted features were clustered using multiple techniques in different configurations. The experiments focus on features directly extracted from the YOLO model, and the effect of the additional dimensionality reduction module is analyzed in later experiments in Section 5.5. The first parameter modified was the layer on the output of the feature extraction block, as shown in Figure 2 and described in Section 3.2. Three different layers were tested to determine whether more complex and higher-dimensional layers would provide an advantage. Another key parameter is the minimum area threshold, which discards small, potentially misleading detections. The proposed threshold skips detections that are lower than a certain percentage of the entire image. The last parameter tested involved the score threshold. Several possible thresholds were tested, but since the main task involves not only detecting people, but also counting them based on features, the effort was made to tailor the chosen threshold to this specific task. MAE was used as the main metric for counting performance, quantifying the average absolute difference between the predicted and actual number of unique people per sequence. For each subset, this metric is computed by comparing the predicted and ground truth counts for each of the nine recordings, and then averaging these differences across all recordings in the subset. It should be emphasized that the MAE metric evaluates the total number of unique individuals across the entire video sequence, rather than the number of people detected in a single frame. Because a sequence naturally accumulates a higher number of unique persons over time, this metric reflects the overall sequence-level counting accuracy rather than frame-level density. In addition, each set of parameters was tested using four different clustering methods. Table 5 shows a comparison of all combinations chosen while using YOLOv10s, revealing that the mean absolute error was the lowest while using the K-means and spherical K-means techniques. Another important aspect is the difference in relation to the parameters. Discarding detections less than 1% of the image resulted in a negative contribution, as did using early layers.

Another factor to consider is the difficulty of each subset. Although each subset includes the same nine rooms, differences arise from the camera used to record the scene, its perspective, and resolution. These factors can influence the difficulty of the task and, consequently, the accuracy of the counting results. Observations presented in Table 6 indicate that the Pi Camera subset is the most challenging, yielding the highest mean absolute errors across all methods and models. This could be attributed to the difficult perspective recorded by the robot, as well as to the higher number of people present in the scene on average. In contrast, the GoPro and Xtion datasets appear to be less complex, resulting in lower error rates. The iPhone and P40Pro subsets exhibit moderate difficulty, with variations depending on the method and model used. Furthermore, comparing the clustering techniques reveals that SK-means tend to perform slightly better than K-means in most cases, particularly for the iPhone and P40Pro subsets. YOLOv10n achieves the best overall performance in the GoPro subset with SK-means, with the lowest MAE of 1.00. To broaden the scope of the evaluation, two established object tracking baselines were also included: DeepSORT [65] and BoT-SORT [66]. Unlike the clustering methods that treat each frame as an independent snapshot, these algorithms utilize temporal consistency by maintaining continuous object trajectories. DeepSORT applies a Kalman filter for motion prediction and the Hungarian algorithm for data association. It further employs a Re-Identification module that extracts appearance embeddings and uses cosine distance for matching detections across frames, enabling robust identity preservation despite occlusions and appearance changes. BoT-SORT operates on a similar principle, combining motion tracking with appearance features, but introduces an additional module for Global Motion Compensation to account for background shifts caused by camera movement.

### 5.5. Encoder Application

The next experiment involved applying the trained encoder as an extension of the proposed method. The primary goal of introducing this additional encoder was to reduce the dimensionality of the features and the further processing time. To train the encoder, the GoPro subset was used as a representative sample of features. The remaining subsets were used for testing to evaluate the impact of embedded features on overall performance. Table 7 presents results for the base YOLOv10n model and two variations with encoder dimensions of 32 (Enc. 32) and 64 (Enc. 64).

A more detailed analysis was conducted, examining the processing times of different stages of the counting pipeline, such as detection, feature extraction, and encoder reduction. In addition, clustering times were evaluated for a selected number of samples. The default model and two different encoders are compared in Table 8. The workstation achieves the lowest processing times, with the fastest setup (YOLOv10n + Enc. 32) reaching a total time of 0.50 s. The mobile workstation is slower, particularly in the clustering steps, where total times range from 1.41 s to 2.26 s, depending on the model and the encoder configuration. The Jetson Nano, being a low-power device, exhibits the longest processing times, exceeding 10 s in most cases, and the YOLOv10n model alone requiring 26.2 s for full processing. These results are characteristic of the computational constraints of the tested platform. Transitioning the proposed framework to newer devices, such as the NVIDIA Jetson Orin series, would significantly improve processing efficiency and further enhance the performance of the system for dynamic robotic applications.

Among the models tested, the base YOLOv10n model without an encoder typically results in longer clustering times, as seen on all platforms. This suggests that while feature encoders add a small per-frame overhead, they improve clustering efficiency, reducing overall computation time for large sequences. Additionally, the choice of encoder size affects performance marginally, with the smaller version generally being slightly faster.

**Figure 8:**
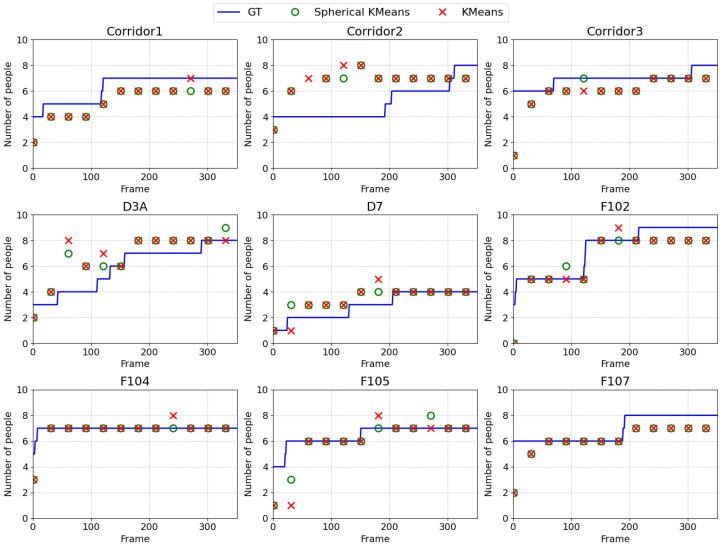
People counting example using YOLOv10n + Enc. 32 on nine P40Pro dataset sequences, with predictions made for every 30 images.

**Figure 9:**
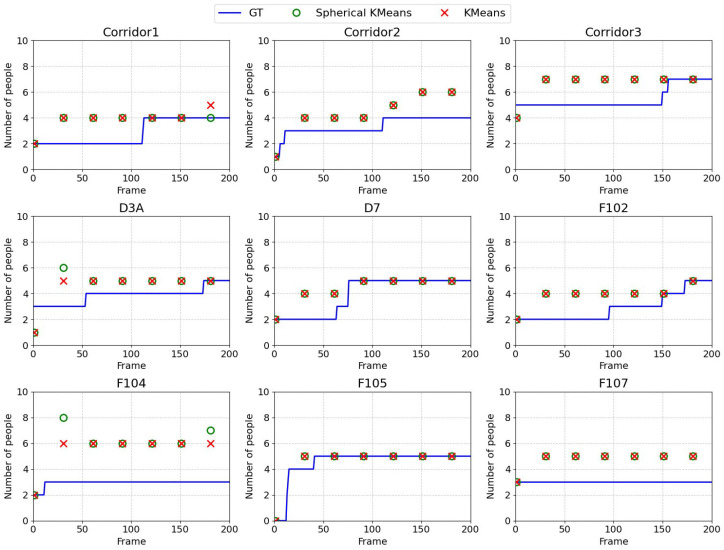
People counting example using YOLOv10n + Enc. 32 on nine Xtion dataset sequences, with predictions made for every 20 images.

An example visualization of the real number of people and the counting results in selected sequence steps is shown in Figure 8 and Figure 9. In these charts, the blue line represents the ground truth (the real number of people in each frame). The green circles denote the predicted number of people at a given moment using Spherical KMeans, while the red crosses represent predictions made using standard KMeans. For each chart, all sequences from the P40Pro and GoPro are presented. For the temporal evaluation, the people count was cumulatively estimated as the sequence progressed. A prediction was generated after every additional 30 images, using all images accumulated from the beginning of the sequence up to that point. The resulting estimate was then compared with the corresponding ground-truth number of people present in the scene. Depending on the sequence, cases can be observed in which the results closely correspond with real data about the number of people present in the scene. Such situations can be seen in the F104 sequence from the P40Pro subset. However, there is the possibility that an error can occur. On the one hand, there are cases where people are overrepresented, which can be seen in the Corridor2 and D7 charts from P40Pro. However, there can be an underrepresentation of the number of people in the scene, for example, Corridor3 from the P40Pro subset.

### 5.6. Additional Model Applications

To evaluate the effectiveness of feature representations beyond those extracted from YOLO models, additional backbones were tested to determine whether changing or combining features could achieve better counting performance. Depending on the backbone, features with different dimensions can be extracted. Lighter versions of YOLO produce lower-dimensional feature maps, whose characteristics can vary depending on the selected layer. This also applies to additional backbones, where models such as SquzeeNet and ResNet-18 extract fewer feature dimensions compared to ViT as presented in Table 9. Several additional backbones of different types were tested and compared with each other. Extracting features independently of YOLO and combining directly affects both the time and the overall accuracy of the results. In this case, the use of selected additional backbone features did not produce a significant improvement in the final error. Across different subsets, minor variations were observed; for example, the ResNet-18 model showed a slight improvement in average MAE (from 1.16 to 1.14), while the use of ViT increased error, raising the average MAE to 1.31.

## 6. Discussion

People counting from mobile robotic platforms presents challenges that differ substantially from those encountered in fixed-camera surveillance systems. In such environments, frequent camera movement, rapidly changing viewpoints, and intermittent visibility of individuals often limit the effectiveness of conventional tracking-based solutions. The findings of this study suggest that sequence-level feature clustering provides a viable alternative by reducing the dependence on persistent trajectories while still allowing accurate estimation of the number of unique individuals observed throughout a sequence.

In our implementation, YOLO is only one component of the proposed detector-agnostic counting framework and was selected because it offers a favorable balance between detection accuracy and computational efficiency, making it well suited for mobile robotic platforms. Although the evaluated YOLO models were trained for general-purpose object detection and not specifically optimized for mobile robot perspectives, they provide a strong baseline despite challenges such as varying viewpoints, motion blur, partial visibility, and frequent occlusions. Future improvements could be achieved by fine-tuning detection models on datasets tailored to mobile robotic people-counting scenarios; however, progress in this direction is currently constrained by the limited availability of large-scale, well-annotated datasets captured from mobile robot viewpoints in indoor environments. The experimental results further indicate that lightweight detector variants, such as YOLOv10n, can provide sufficiently discriminative features for accurate sequence-level counting while maintaining low computational requirements, making them particularly attractive for deployment on resource-constrained mobile robots.

The encoder-based dimensionality reduction introduced an important trade-off between computational efficiency and counting accuracy. Although the encoder slightly increased the average MAE in some configurations, it significantly reduced clustering time, especially on the Jetson Nano platform, where the total processing time decreased from approximately 26 s to about 10 s for a 500-frame sequence. Therefore, the encoder can be considered a practical optimization mechanism for resource-constrained systems, where computational efficiency may be more critical than achieving the lowest possible counting error.

The analysis of individual subsets revealed that recordings obtained from the Pi Camera robot were the most challenging due to the low camera position, changing viewpoints, partial visibility of people, and frequent occlusions. These factors affected both detection quality and feature consistency used during clustering. Failure cases in this subset typically arise when individuals are partially obscured by environmental obstacles or each other, causing fragmented feature clusters and incorrect identification. These results demonstrate that while the proposed framework is robust, its accuracy is inherently linked to viewpoint visibility, motivating future research into viewpoint-invariant feature learning for mobile robots. Although the evaluation was limited to indoor environments and relatively small groups of people, the obtained results indicate that the proposed combination of YOLO-based detection and feature clustering can serve as an effective solution for people counting on mobile robotic platforms. However, it is important to note that this bounding-box and clustering approach is inherently tailored for environments with a limited number of individuals. In scenarios involving dense crowds, severe occlusions and overlapping individuals would significantly degrade both the YOLO detector’s performance and the quality of the extracted features. For such large-scale crowd scenarios, regardless of whether they are indoor or outdoor, density map estimation techniques are generally more appropriate and effective than instance-level detection and clustering [67,68]. Furthermore, while existing tracking and re-identification frameworks are powerful, they are typically optimized for static viewpoints or continuous temporal data [65,66]. This presents significant methodological challenges when applying them to the dynamic camera movements and discontinuous observations inherent to our mobile robotic platform. In contrast, our clustering-based formulation avoids reliance on persistent trajectories, which allows the system to remain robust in the face of frequent track fragmentation and more effectively handle repeated person appearances across the sequence.

## 7. Conclusions

In this paper, we have presented a novel approach to visual people counting in dynamic environments using images also recorded by mobile robots. The proposed method combines YOLO-based people detection with feature clustering to estimate the number of individuals in an image sequence. During the experiments, different parameters, such as the backbone layer from which features were extracted, the confidence score threshold, and the minimal ROI area, were compared, and the best configuration was selected. Three variants of YOLOv10 were compared, and the nano version was selected for the remaining experiments due to its good performance and low inference time. Each subset was tested separately to evaluate performance from a varying perspective. Particularly challenging were the Pi Camera subset data, which represent views from a difficult camera perspective on the robot. In such scenarios, the camera can often only capture the lower parts of the human body, making accurate detection more difficult. The method utilizes an encoder, which was compared in two variants and proved beneficial in improving clustering efficiency and reducing computational costs, particularly on resource-limited platforms such as Jetson Nano. An evaluation on a dataset with four different clustering methods demonstrated the effectiveness of the approach. The best results were achieved using YOLOv10n alongside K-means and SK-means clustering. These results highlight the potential of integrating deep learning-based detection with feature clustering for real-time people counting in mobile robotic applications. This work and the collected dataset may serve as a foundation for future research and development in vision-based people detection, re-identification, and counting. Future efforts will focus on transitioning the proposed framework to real-time online deployment on mobile robotic platforms. By leveraging advanced hardware, such as the Jetson Orin series, we aim to further enhance computational efficiency and facilitate integration in dynamic, real-world environments.

## Figures and Tables

**Table 1:** Properties of the used subsets.

Subset	Camera	Resolution [px]	Aspect Ratio	mFPS	No. Images	No. BBox
iPhone	Human	960 × 540	1.7	8.8	4500	8405
GoPro	Human	960 × 540	1.7	9.3	4500	6480
P40Pro	Human	640 × 360	1.7	4.3	3150	6849
Pi Camera	Robot	640 × 480	1.3	6.0	5400	8817
Xtion	Robot	640 × 480	1.3	1.3	1800	1843

**Table 2:** Average number of bounding boxes (number of persons) per image for subsets.

Camera	Corridor1	Corridor2	Corridor3	D3A	D7	F102	F104	F105	F107
GoPro	1.88	1.71	1.15	0.93	0.79	2.10	2.80	2.48	2.98
iPhone	1.73	1.28	1.07	0.26	0.74	1.32	2.16	1.63	2.77
P40Pro	1.70	2.42	2.74	1.97	0.81	2.44	2.72	1.40	3.34
Pi Camera	1.70	2.89	2.72	1.52	0.56	1.05	1.67	0.90	1.70
Xtion	0.64	1.63	2.20	1.11	0.53	0.74	1.03	0.52	0.84
Avg.	1.73	1.99	1.98	1.36	0.69	1.53	2.08	1.39	2.33

**Table 3:** Specifications of the platforms used in the experiments.

Feature	Platform		
	Mobile Workstation	Workstation	Jetson Nano
Operating System	Windows	Ubuntu 22.04 LTS	Ubuntu 20.04
CPU Model	Intel Core i7-11800H	AMD Ryzen Threadripper 3970X	ARM Cortex-A57
CPU Clock Speed/Turbo	2.3/4.6 GHz	3.7/4.5 GHz	1.43 GHz
CPU Cores/Threads	8/16	32/64	4
RAM	16 GB	64 GB	4 GB
GPU Model	NVIDIA GeForce RTX 3060 M	NVIDIA GeForce RTX 2080 Ti	NVIDIA Maxwell GPU
GPU Memory	6 GB	11 GB	0.5 GB
GPU Cores	3840	4352	128
GPU Clock Speed	1.6 GHz	2.2 GHz	0.8 GHz
GPU Architecture	Ampere	Turing	Maxwell
AI Performance	10.9 TFLOPS	13.5 TFLOPS	0.5 TFLOPS
Python Version	3.8	3.10	3.8
PyTorch Version	2.2	2.2	1.13
Compute Capability	8.6	7.5	5.3
CUDA Version	11.8	12.4	10.8

**Table 4:** Comparison of different detection methods. The best score is bolded, while the second-best score is underlined.

Model	Backbone	Params	Input Size [px]	AP50 [%]	AP50:95 [%]	F1-Score [%]	Time [ms] *
DETR [27]	ResNet-50	41.3 M	640 × 640	71.29	51.99	80.42	35.90
			320 × 320	61.47	36.67	72.97	31.43
FasterRCNN [26]	ResNet-50FPN	43.7 M	640 × 640	73.87	54.10	81.53	66.41
			320 × 320	70.03	50.36	79.24	48.99
FCOS [64]	ResNet-50FPN	32.3 M	640 × 640	63.81	44.21	75.24	42.00
			320 × 320	42.55	29.08	57.49	38.88
RT-DETR-L [63]	CSPResNet-50	33.0 M	640 × 640	**80.08**	**65.41**	**85.87**	42.52
			320 × 320	74.58	55.77	82.49	30.26
RetinaNet [59]	ResNet-50FPN	38.2 M	640 × 640	60.29	42.23	71.83	47.59
			320 × 320	51.15	34.39	65.07	27.89
YOLOXm [60]	CSPDarknet53	25.3 M	640 × 640	65.59	47.85	76.46	22.26
			320 × 320	62.68	44.16	74.09	21.16
YOLOv5m [61]	CSPDarknet53	21.2 M	640 × 640	74.27	56.30	82.73	20.97
			320 × 320	74.66	56.14	82.84	**14.63**
YOLOv8m [62]	CSPDarknet53	25.9 M	640 × 640	79.84	63.41	85.33	29.15
			320 × 320	77.82	61.73	84.29	17.78
YOLOv10m [50]	CSPDarknet53	15.4 M	640 × 640	78.06	62.50	84.67	23.51
			320 × 320	75.00	59.35	82.56	14.72

* Time for a Mobile Workstation.

**Table 5:** Comparison of YOLOv10s with varying parameters configurations. The table presents the MAE for people counting for different clustering techniques based on model configurations. Bold values indicate the best result.

	Model Configuration			MAE			
**Model**	**Block**	**Min. ROI [px%]**	**Score Thr.**	**K-Means**	**Mean-Shift**	**DBSCAN**	**SK-Means**
YOLOv10s	15	1	0.7	1.47	2.00	2.58	1.31
	20	1	0.7	1.36	1.96	2.00	1.29
	21	1	0.7	**1.27**	1.84	2.29	1.29
	21	2	0.7	1.36	1.93	2.27	1.62
	21	4	0.7	1.42	1.93	2.22	**1.20**
	21	1	0.5	1.31	1.87	**1.96**	1.51
	21	1	0.6	**1.27**	**1.78**	2.11	1.44
	21	4	0.8	1.38	2.07	2.53	1.51

**Table 6:** Comparison of people counting results for different subsets, best results are bolded.

		MAE					
**Model**	**Method**	**GoPro**	**iPhone**	**P40Pro**	**Pi Camera**	**Xtion**	**Avg.**
YOLOv10n	K-means	1.44	1.11	0.67	**1.55**	1.11	**1.18**
YOLOv10s		1.15	1.11	0.88	1.89	**0.89**	1.26
YOLOv10m		1.11	1.22	1.44	2.11	1.22	1.42
YOLOv10n	SK-means	1.22	**0.89**	**0.33**	2.33	**0.89**	**1.13**
YOLOv10s		1.22	1.77	1.00	2.33	**0.89**	1.44
YOLOv10m		**1.00**	1.22	1.00	2.11	1.33	1.33
YOLOv10n	DeepSORT [65]	1.78	3.00	5.22	2.89	1.56	2.89
YOLOv10n	BoT-SORT [66]	3.78	4.22	3.33	4.89	2.78	3.80

**Table 7:** Comparison of people counting results with different encoder variants, best results are bolded.

		MAE *				
**Model**	**Clustering Method**	**iPhone**	**P40Pro**	**Pi Camera**	**Xtion**	**Avg.**
YOLOv10n	K-means	1.11	0.67	**1.55**	1.11	**1.11**
YOLOv10n + Enc. 32		1.00	0.67	2.22	**0.78**	1.16
YOLOv10n + Enc. 64		1.11	0.77	2.22	**0.78**	1.22
YOLOv10n	SK-means	**0.89**	0.33	2.33	0.89	**1.11**
YOLOv10n + Enc. 32		1.00	0.56	2.22	0.89	1.16
YOLOv10n + Enc. 64		1.11	**0.66**	2.22	1.00	1.25

* GoPro was used to train the encoder.

**Table 8:** Processing times from sample sequence with different hardware configurations and methods, where the lowest total times are highlighted in bold.

Hardware	Model	Per-Frame [s]				Clustering [s]			Total * [s]
		Detection	Extraction	Encoder	Det. + Ex. + En.	100	300	500	
Mobile workstation	YOLOv10n	2.63 × $10^{-2}$	0.70 × $10^{-3}$	—	2.70 × $10^{-2}$	1.24	1.36	2.23	2.26
	YOLOv10n + Enc. 32	2.51 × $10^{-2}$	0.41 × $10^{-3}$	1.52 × $10^{-3}$	2.70 × $10^{-2}$	1.14	1.23	1.38	**1.41**
	YOLOv10n + Enc. 64	2.61 × $10^{-2}$	0.45 × $10^{-3}$	1.45 × $10^{-3}$	2.80 × $10^{-2}$	1.17	1.31	1.57	1.60
Workstation	YOLOv10n	2.12 × $10^{-2}$	0.22 × $10^{-3}$	—	2.15 × $10^{-2}$	0.28	0.67	0.96	0.98
	YOLOv10n + Enc. 32	1.86 × $10^{-2}$	0.22 × $10^{-3}$	1.03 × $10^{-3}$	1.99 × $10^{-2}$	0.22	0.29	0.48	**0.50**
	YOLOv10n + Enc. 64	2.08 × $10^{-2}$	0.21 × $10^{-3}$	1.05 × $10^{-3}$	2.20 × $10^{-2}$	0.23	0.45	0.57	0.59
Jetson Nano	YOLOv10n	1.36 × $10^{-1}$	1.53 × $10^{-3}$	—	1.37 × $10^{-1}$	7.25	2.30 × 10	2.61 × 10	**2.62 × 10**
	YOLOv10n + Enc. 32	1.48 × $10^{-1}$	1.72 × $10^{-3}$	5.13 × $10^{-3}$	1.55 × $10^{-1}$	1.75	3.56	9.94	1.01 × 10
	YOLOv10n + Enc. 64	1.88 × $10^{-1}$	4.34 × $10^{-3}$	5.42 × $10^{-3}$	1.98 × $10^{-1}$	2.97	7.73	1.04 × 10	1.06 × 10

* Total latency calculated specifically for the 500-frame mark, representing the sum of a single frame’s processing time (Det. + Ex. + En.) and the clustering execution time over 500 accumulated features.

**Table 9:** Comparison of results with additional features with YOLOv10n as base detector and an Enc. 64, lowest average MAE highlighted in bold.

Model	Additional Model	Feature Size *	MAE **				
			iPhone	P40Pro	Pi Camera	Xtion	Avg.
YOLOv10n	–	384	1.00	0.67	2.22	0.78	1.16
YOLOv10n	Mobilenetv3	384 + 576	1.00	0.67	2.22	0.78	1.17
YOLOv10n	ResNet-18	384 + 512	1.00	0.56	2.22	0.78	**1.14**
YOLOv10n	SqueezeNet	384 + 512	1.00	0.56	2.33	0.78	1.17
YOLOv10n	ViT	384 + 768	1.11	0.78	2.22	1.11	1.31

* Feature size before dimension reduction; ** The GoPro subset was used to train the encoder.

## Data Availability

The datasets, annotations, and source code used in this study are publicly available on the GitHub platform at https://github.com/GomulkaK/PeopleCounting [55].
